# Minimally Invasive Pancreaticoduodenectomy in Elderly versus Younger Patients: A Meta-Analysis

**DOI:** 10.3390/cancers16020323

**Published:** 2024-01-11

**Authors:** Roberto Ballarin, Giuseppe Esposito, Gian Piero Guerrini, Paolo Magistri, Barbara Catellani, Cristiano Guidetti, Stefano Di Sandro, Fabrizio Di Benedetto

**Affiliations:** Hepato-Pancreato-Biliary Surgery and Liver Transplantation Unit, Policlinico Modena Hospital, Azienda Ospedaliero Universitaria di Modena, Via del Pozzo 71, 41125 Modena, Italy; giuseppe.esposito@unimore.it (G.E.); guerrinigp@yahoo.it (G.P.G.); paolo.magistri@unimore.it (P.M.); catellani.barbara@aou.mo.it (B.C.); cguidetti@unimore.it (C.G.);

**Keywords:** pancreaticoduodenectomy, elderly patients, minimally invasive surgery, minimally invasive pancreaticoduodenectomy, robotic pancreaticoduodenectomy, laparoscopic pancreaticoduodenectomy

## Abstract

**Simple Summary:**

With ageing, the number of pancreaticoduodenectomies (PD) for benign or malignant disease is expected to increase in elderly patients. The aim of the present meta-analysis is to compare the surgical outcomes of MIPD in elderly versus younger patients. The results of our analysis disclose no considerable differences in terms of technical and post-operative outcomes between the two groups. However, slightly higher, but acceptable, major complication and mortality rates were recorded in the elderly cohort. Although the real additional value of minimally invasive surgery in this frailty subset of patients needs to be further investigated, our findings reveal that MIPD seems to be relatively safe and feasible in elderly patients.

**Abstract:**

(1) Background: With ageing, the number of pancreaticoduodenectomies (PD) for benign or malignant disease is expected to increase in elderly patients. However, whether minimally invasive pancreaticoduodenectomy (MIPD) should be performed in the elderly is not clear yet and it is still debated. (2) Materials and Methods: A systematic review and meta-analysis was conducted including seven published articles comparing the technical and post-operative outcomes of MIPD in elderly versus younger patients up to December 2022. (3) Results: In total, 1378 patients were included in the meta-analysis. In term of overall and Clavien–Dindo I/II complication rates, post-operative pancreatic fistula (POPF) grade > A rates and biliary leakage, abdominal collection, post-operative bleeding and delayed gastric emptying rates, no differences emerged between the two groups. However, this study showed slightly higher intraoperative blood loss [MD 43.41, (95%CI 14.45, 72.38) *p* = 0.003], Clavien–Dindo ≥ III complication rates [OR 1.87, (95%CI 1.13, 3.11) *p* = 0.02] and mortality rates [OR 2.61, (95%CI 1.20, 5.68) *p* = 0.02] in the elderly compared with the younger group. Interestingly, as a minor endpoint, no differences in terms of the mean number of harvested lymphnode and of R0 resection rates were found. (4) Conclusion: MIPD seems to be relatively safe; however, there are slightly higher major morbidity, lung complication and mortality rates in elderly patients, who potentially represent the individuals that may benefit the most from the minimally invasive approach.

## 1. Introduction

Over the past few years, the incidence of pancreatic and periampullary tumors has continuously increased with ageing; thus, the number of pancreaticoduodenectomies (PD) is expected to increase in elderly patients, as radical pancreatic resection is the only potentially curative treatment for pancreatic and periampullary malignancies [1,2]. Particularly, pancreatic adenocarcinoma accounts for 6% of all cancer-related deaths, and major morbidity related to pancreatic surgery still occurs in 40% of patients, with a mortality rate ranging between 2% and 5% [3]. For a long time, being older than 75 years has been considered a limitation for pancreatic surgery due to concomitant comorbidities. However, with adequate patient selection, preoperative supportive care and advances in surgical skills and techniques, age should no longer be considered a contraindication for PD [2,4]. In 1994, Gagner et al. [5] described the first laparoscopic PD (LPD) and, in 2003, Giulianotti et al. reported the first robotic PD (RPD) [6]. Since then, the use of minimally invasive PDs (MIPD) is growing worldwide. However, both laparoscopic and robotic pancreatic surgeries still represent a small fraction of PDs and have not yet been considered as a standard alternative to open PD [7]. MIPD has been demonstrated to be safe and feasible in well-selected patients in high volume centers [8]. Minimally invasive surgery (MIS) offers significant advantages over the open approach in terms of intraoperative and postoperative outcomes, as well as faster recovery [9]. Moreover, several studies have reported that LPD achieves acceptable surgical and oncological outcomes in elderly patients [4,10]. Furthermore, RPD has been recently demonstrated to overcome some of the shortcomings of laparoscopic surgery. However, whether the robotic approach should be performed in the elderly is not clear yet and it is still being debated [4,11,12]. The primary aim of this meta-analysis is to compare the surgical intra- and post-operative outcomes in elderly and younger patients for MIPD by including all the existing observational clinical studies on this topic from the current literature. Additionally, the oncological safety of MIPD is a secondary endpoint of the analysis.

## 2. Materials and Methods

### 2.1. Study Design

Our meta-analysis was designed according to the Preferred Reporting Items for Systematic Reviews and Meta-analyses (PRISMA) statement [13]; however, this meta-analysis was not registered in the Prospective Register of Systematic Reviews (PROSPERO). The authors predetermined the eligibility criteria for the study, and two investigators (G.E. and R.B.) independently searched the literature. All retrospective clinical studies that compared MIPD in elderly with MIPD in young patients were included in the present meta-analysis. Until now, no prospective or randomized controlled trials have been published on this topic. Case reports, letters and reviews were excluded. Any discrepancies identified during the data collection, synthesis and analysis were discussed and resolved through consensus between two authors (G.E. and R.B.). The PRISMA [13] and MOOSE [14] checklists are reported in Supplementary Files S1 and S2, respectively.

### 2.2. Literature Search and Data Collection

We conducted a systematic search of the literature in the PubMed, MEDLINE and Cochrane library databases for articles published up to December 2022. As stated by Goossen et al., we queried three databases to maximize the likelihood of capturing relevant articles [15]. Our search included the words “pancreaticoduodenectomy”, “robotic pancreaticoduodenectomy”, “laparoscopic pancreaticoduodenectomy”, “minimally invasive pancreaticoduodenectomy” and “pancreaticoduodenectomy and elderly and young”. The search strategy was confined to English language articles and is described in Supplementary File S3.

### 2.3. Quality Assessment

The quality of the included studies was assessed with the Methodological Index for Non-Randomized Studies (MINORS) [16].

### 2.4. Statistical Analysis

Meta-analysis was performed using the software Review Manager (RevMan) [Version 5.1. Copenhagen: The Nordic Cochrane Centre, The Cochrane Collaboration, 2011). Dichotomous variables are reported as odds ratios (OR) with 95% confidence interval (CI) by using the Mantel–Haenszel method and continuous outcomes as Mean difference (MD) with 95% CI by utilizing the generic inverse variance method. Mean and standard deviation (SD) for continuous data, if not reported, were estimated using the method illustrated by Hozo et al. [17]. However, for continuous data provided as median and interquartile range (IQR) mean and SD were estimated with the method described by Luo et al. [18] and Wan et al. [19], respectively. The cut-off for statistical significance was set at *p* ≤ 0.05. Heterogeneities between the studies were evaluated using Q statistics and total variation was computed using I^2^ [20]. A random-effects model (REM) was always adopted due to the conceptual heterogeneity of clinical studies. Publication biases of the included papers are reported in Supplementary File S4. The patients and tumor characteristics of the individual studies are summarized in Supplementary File S5.

## 3. Results

### 3.1. Studies and Patient Characteristics

**Figure 1:**
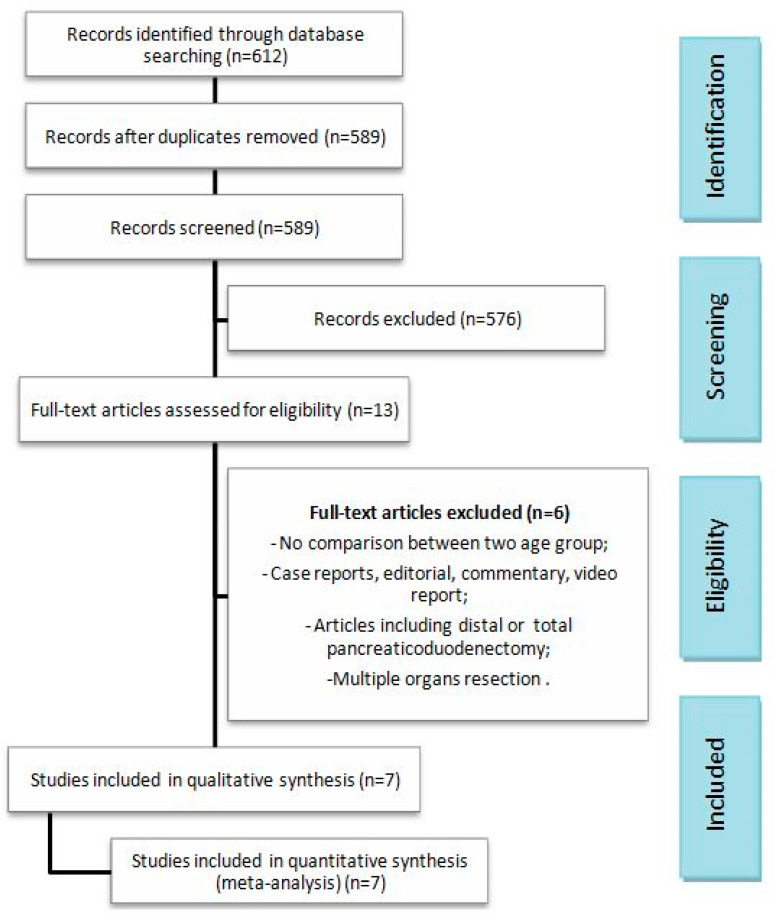
PRISMA flow diagram.

The search strategy disclosed 589 publications concerning MIPD. Thirteen full papers were retrieved; among these, six studies were not included in the analysis due to missing inclusion criteria. Finally, 7 articles and a total of 1378 subjects were included in the meta-analysis: 326 were elderly patients and 1016 were relatively young individuals [4,10,21,22,23,24,25]. Non-randomized control trials were included in the meta-analysis. The search process is displayed in the PRISMA flow diagram in Figure 1. Letters, reviews, comments, posters and protocols were excluded. The baseline features of the included studies and of the two groups are presented in Table 1 and Table 2. Post-operative and technical outcomes are tabulated in Table 3. The two groups were similar regarding gender, BMI, pre-operative biliary drainage, CA 19.9 and tumor diameter. As expected, age, overall comorbidities, in particular hypertension, and the American Society of Anesthesiologist (ASA) scores differ between the two groups, indeed this is related to the included studies’ design. The number of enrolled patients in each study ranged from a minimum of 41 up to 431. The MINORS scale assessed a low-quality heterogeneity between studies, providing a mean score of 22.3 (SD: 0.76) and a median score of 22 (range 21–23) (Table 1).

### 3.2. Technical and Post-Operative Outcomes

#### 3.2.1. Operating Time

**Figure 2:**
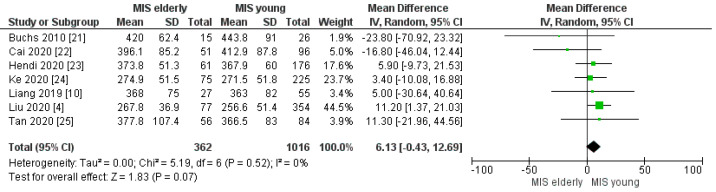
Operating time (min) [4,10,21,22,23,24,25].

The mean operating time was 354.06 min in the MIS*e* group and 354.49 min in the MIS*y* group; seven articles reported the duration of surgery. The operating time was similar between the two groups and the meta-analysis revealed no statistically significant difference [MD 6.13, (95%CI −0.43, 12.69) *p* = 0.07], Figure 2.

#### 3.2.2. Intraoperative Blood Loss and Intraoperative Red Blood Cell (RBC) Transfusion Rate

**Figure 3:**
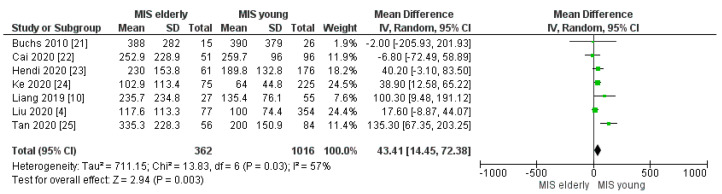
Intraoperative blood loss (mL) [4,10,21,22,23,24,25].

**Figure 4:**
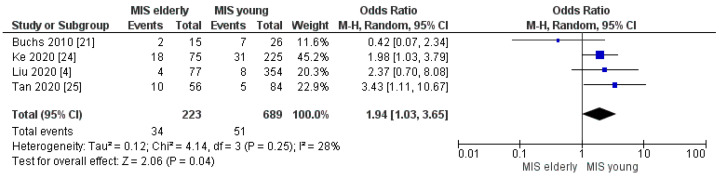
Intraoperative Transfusion rate [4,21,24,25].

Our meta-analysis showed statistically significant increased intraoperative blood loss and RBC transfusion rates in the elderly group when compared with the younger one [MD 43.41, (95%CI 14.45, 72.38) *p* = 0.003] and [OR 1.94, (95%CI 1.03, 3.65) *p* = 0.04], respectively. The mean estimated blood loss was 237.49 cc in the elderly cohort and 191.27 cc in the young cohort, Figure 3. Indeed, the mean intraoperative transfusion rates in the elderly and young groups were 15% and 7% Figure 4, respectively. 

#### 3.2.3. Conversion to Open Surgery and Reoperation Rate

**Figure 5:**
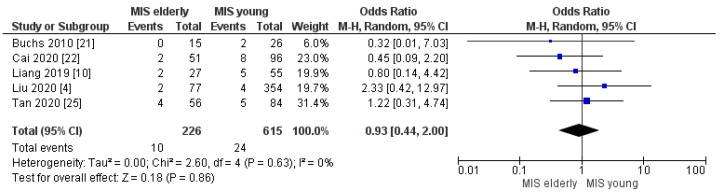
Conversion to Open rate [4,10,21,22,25].

**Figure 6:**
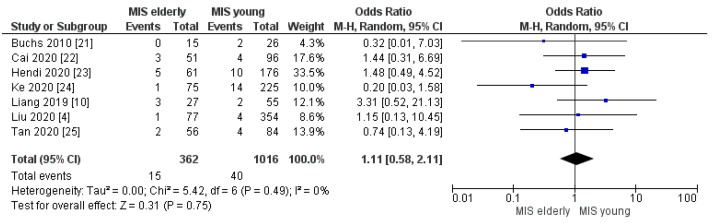
Reoperation rate [4,10,21,22,23,24,25].

Conversion to open surgery and reoperation rates resulted as being equivalent between the two groups and the meta-analysis disclosed no statistically significant difference in the rate of the two items, [OR 0.93, (95%CI 0.44, 2.00) *p* = 0.86] and [OR 1.11, (95%CI 0.58, 2.11) *p* = 0.75], Figure 5 and Figure 6, respectively. The conversion rate was 4.0% (10/226) in the elderly and 4.0% (24/615) in the young group. The reoperation rate was 4.0% (15/362) in MIS*e* and 4.0% (40/1016) in MIS*y*.

#### 3.2.4. Peri-Operative Mortality Rate

**Figure 7:**
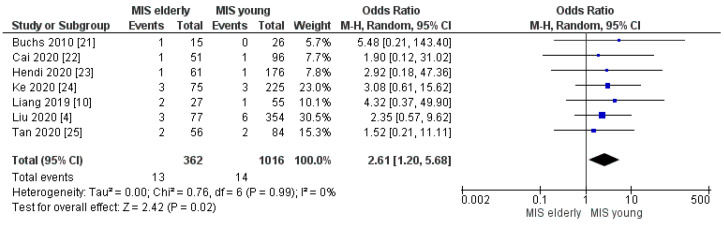
Perioperative Mortality rate [4,10,21,22,23,24,25].

The meta-analysis of the seven studies showed a statistically significant difference in the rates of perioperative mortality between the two groups [OR 2.61, (95%CI 1.20, 5.68) *p* = 0.02], Figure 7. The mortality rate resulted as 4.0% (13/362) in the MIS*e* group and 1.0% (14/1016) in the MIS*y* group; therefore, it was slightly higher in the elderly cohort. 

#### 3.2.5. Complication Rate

**Figure 8:**
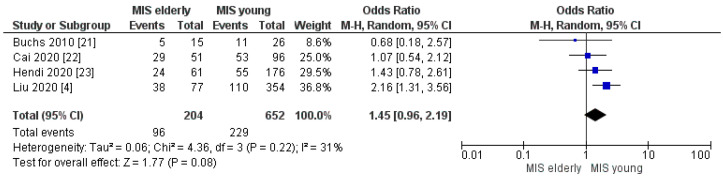
Overall Complication rate [4,21,22,23].

**Figure 9:**
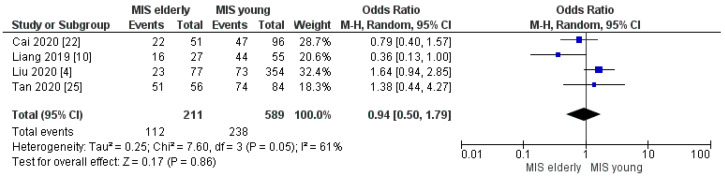
Clavien–Dindo I/II rate [4,10,22,25].

**Figure 10:**
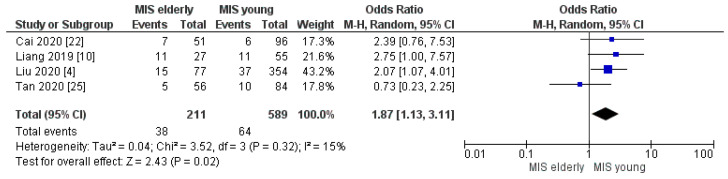
Clavien–Dindo ≥ III rate [4,10,22,25].

Four trials assessed the postoperative complication rates according to the Clavien–Dindo classification. The meta-analysis disclosed no significant difference in the rates of overall morbidity and of Clavien–Dindo I/II complications between the two groups. However, a slightly higher morbidity rate was disclosed in the elderly cohort: MIS*e* 47% (96/204) and MIS*y* 35% (229/652), [OR 1.45, (95%CI 0.96, 2.19) *p* = 0.08], Figure 8; and MIS*e* 53% (112/211) and MIS*y* 40% (238/589), [OR 0.94, (95%CI 0.50, 1.79) *p* = 0.86], Figure 9, respectively. On the other hand, the meta-analysis showed a statistically significant difference in the Clavien–Dindo ≥ III complication rates with higher morbidity in the MIS*e* group than in the younger one: 18% (38/211) and 11% (64/589), [OR 1.87, (95%CI 1.13, 3.11) *p* = 0.02], Figure 10, respectively.

#### 3.2.6. Post-Operative Pancreatic Fistula Grade > A and Biliary Leakage Rate

**Figure 11:**
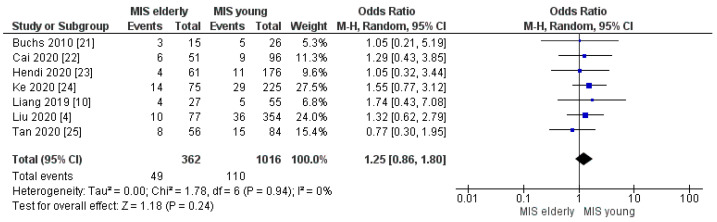
POPF grade > A rate [4,10,21,22,23,24,25].

**Figure 12:**
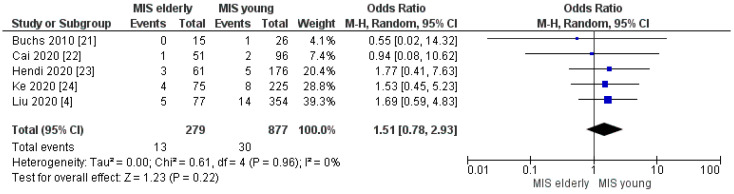
Biliary Leakage rate [4,21,22,23,24].

A total of 159 patients developed a POPF grade > A. The POPF > A rate was 14% (49/362) in the MIS*e* group and 11% (110/1016) in the MIS*y* group. The meta-analysis did not show a significant difference in the rate of pancreatic leakage between the two groups [OR 1.25, (95%CI 0.86, 1.80) *p* = 0.24], Figure 11. Five studies reported the frequency of biliary fistula. The biliary leakage rate was similar between the two groups with no statistically significant difference: MIS*e* 5% (13/279) and MIS*y* 3% (30/877), [OR 1.51, (95%CI 0.78, 2.93) *p* =0.22], Figure 12.

#### 3.2.7. Post-Op Bleeding, Delayed Gastric Empty and Abdominal Collection Rates

**Figure 13:**
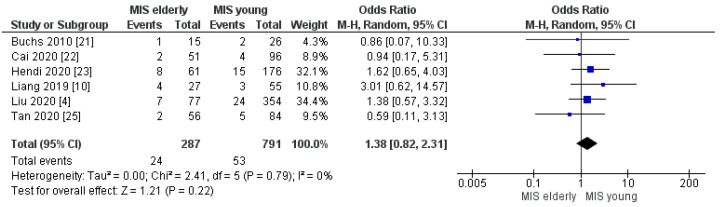
Postoperative Bleeding rate [4,10,21,22,23,25].

**Figure 14:**
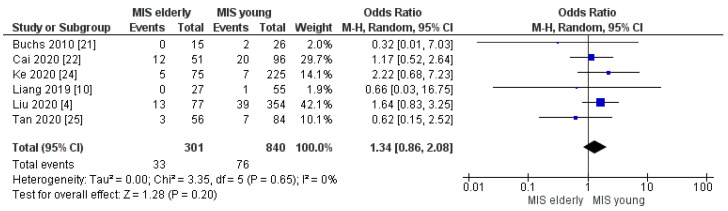
Delayed Gastric Empty rate [4,10,21,22,24,25].

**Figure 15:**
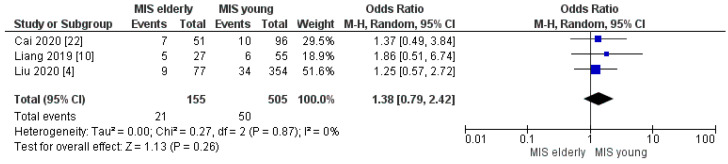
Abdominal Collection rate [4,10,22].

Three and six papers assessed the abdominal collection, the post-operative bleeding and the delayed gastric empty (DGE) rates, respectively. The meta-analysis disclosed no statistically significant difference in the rates of these morbidities between the two groups, with similar bleeding and DGE rates in the MIS*e* and MIS*y* cohorts: MIS*e* 8% (24/287) and MIS*y* 7% (53/791), [OR 1.38, (95%CI 0.82, 2.31) *p* = 0.22], Figure 13; and MIS*e* 11% (33/301) and MIS*y* 9% (76/840), [OR 1.34, (95%CI 0.86, 2.08) *p* =0.20], Figure 14, respectively. Though the abdominal collection rate resulted as being slightly higher in the elderly group than the young group, this finding was not statistically significant, MIS*e* 14% (21/155) and MIS*y* 10% (50/505), [OR 1.38, (95%CI 0.79, 2.42) *p* = 0.26], Figure 15.

#### 3.2.8. Lung Morbidity Rate

**Figure 16:**
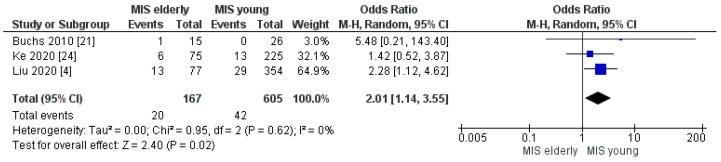
Lung Morbidity rate [4,21,24].

Only three papers reported the lung complication rates. However, perioperative lung morbidity rates resulted as 12% (20/167) in the MIS*e* group and 7% (42/605) in the MIS*y* group. The meta-analysis showed that the pulmonary complication rate was significantly higher in the MIS*e* than the MIS*y* group, [OR 2.01, (95%CI 1.14, 3.55) *p* = 0.02], Figure 16.

#### 3.2.9. R0 Margin Rate and Mean Number of Harvested Lymphnodes

**Figure 17:**
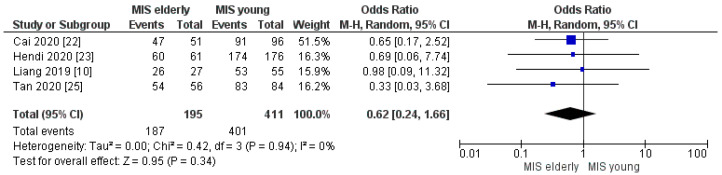
R0 Margin rate [10,22,23,25].

**Figure 18:**
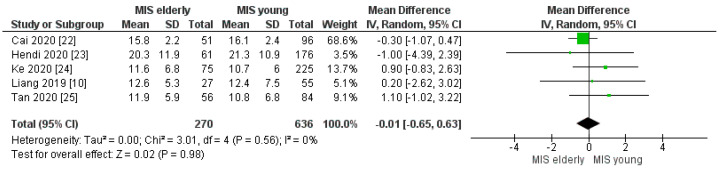
Number of harvested lymphnodes [10,22,23,24,25].

Four of the seven studies reported the R0 margin rate. The R0 margin rate was 96% (187/195) in the MIS*e* group and 98% (401/411) in the MIS*y* group, but with no statistically significant difference between the two cohorts [OR 0.62, (95%CI 0.24, 1.66) *p* = 0.34], Figure 17. Moreover, our meta-analysis did not reveal a statistically significant difference in the number of harvested lymphnodes in the MIS*e* group when compared with the MIS*y* group, [MD −0.01, (95%CI −0.65, 0.63) *p* = 0.98], Figure 18. The mean number of retrieved nodes in the elderly and young groups was 14.4 and 14.3, respectively.

#### 3.2.10. Readmission Rate

**Figure 19:**
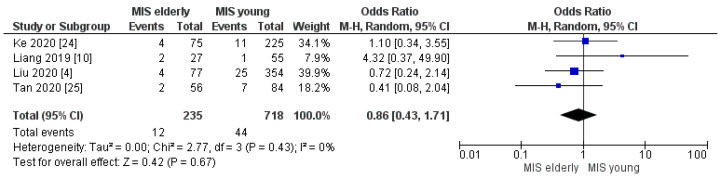
Readmission rate [4,10,24,25].

The readmission rate was 5% (12/235) in the elderly group, and 6% (44/718) in the young sample. The meta-analysis showed no statistically significant difference between the two groups, [OR 0.86, (95%CI 0.43, 1.71) *p* = 0.67], Figure 19.

#### 3.2.11. Length of Hospital Stay 

**Figure 20:**
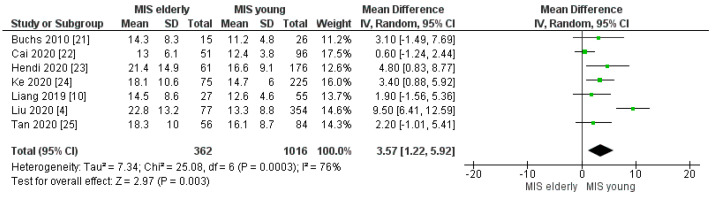
Length of hospital stay [4,10,21,22,23,24,25].

All included studies reported the length of hospital stay. The mean hospital stay was 17.5 days in the MIS*e* group and 13.8 in the MIS*y* group. The meta-analysis disclosed that the mean hospital stay was significantly longer in the MIS*e* group than in the MIS*y* group, [MD 3.57, (95%CI 1.22 5.92) *p* =0.003], Figure 20.

## 4. Discussion

The elderly and very elderly populations are expected to increase in the coming years thanks to the progressions in public health and medical care. As a consequence, surgeons will face a higher number of elderly patients being diagnosed with pancreatic cancer. PD still represents the only potentially curative treatment for PC, and an uneventful and fast recovery after pancreatic surgery is pivotal to initiating the adjuvant therapies without delay and discontinuation [3]. Advanced age and diminished physiological reserve impair the ability to recover and to withstand a major operation; moreover, some authors disclosed an increased morbidity rate, mainly consisting of cardiopulmonary complications [22]. MIS achieves a faster recovery by reducing surgical-related stress and immunological trauma. Moreover, RPD seems to offer some additional advantages in terms of lower conversion to open and transfusion rates compared with LPD, although both approaches appear to achieve equivalent clinical and post-operative outcomes. However, whether the application of the laparoscopy or the robot-method has any additional values for elderly patients is still debated and needs to be further investigated [26]. Therefore, the aim of this study is to evaluate the safety and feasibility of MIPD in elderly people compared with younger patients.

Our meta-analysis revealed a significantly increased mean intraoperative blood loss in the elderly group compared with the younger one, 237.49 cc and 191.27 cc, respectively, with a higher red blood cell transfusion rate in the elder cohort than in the younger patients (15% vs. 7%). Therefore, MIPD does not perform as well in elderly patients as it does in the younger group, and apparently it does not add any particular advantage. However, these differences could be explained by the unavoidable weakness related to tissue texture changes in organs, the wide use of anticoagulant or antiplatelet therapies and the reduced tolerance to ischemia in elderly patients [10,24,27]. Nevertheless, it has also been largely demonstrated that MIS decreases the intraoperative blood loss and transfusion need, thanks to the more precise organ dissection and vessel identification, compared with the open approach.

In terms of operating time, conversion to open rates and re-operation rates, no differences emerged from our analysis between the two groups. Historically, the conversion rate during MIPS has been reported to be as high as 30% [22]. The most common reasons for conversion during PD were locally advanced tumors with the involvement of vascular structures requiring resection and reconstruction and uncontrollable bleeding [4]. On the other hand, abdominal infection, grade C post-operative pancreatic fistula (POPF), the redoing of the hepaticojejunostomy and arterial hemostasis were the main indications for re-operation [21]. Therefore, operating time and conversion and re-operation rates resulted as similar in elderly and younger patients, and this may be due to the fact that the studies included in the meta-analysis have been conducted in high volume centers for MIPS and after achieving the learning curve of this technically demanding operation [4,28]. Therefore, it can be assumed that these factors are more related to technical issues and surgical skills than to patients’ characteristics.

Interestingly, the meta-analysis disclosed no differences in terms of overall and Clavien–Dindo I/II complication rates. No significant differences were also recorded in the rates of POPF grade > A, biliary leakage, abdominal collection, post-operative bleeding and delayed gastric emptying between the two groups. However, the Clavien–Dindo ≥ III complication rate and the lung morbidity rate resulted as significantly higher in the elderly group (MIS*e*) compared with the younger one (MIS*y*), with an 18% (range, 6.6–20%) and 11% (range, 6.2–19.2%) Clavien–Dindo ≥ III morbidity rate and a 12% and 7% pulmonary complication rate in the MIS*e* and MIS*y* groups, respectively. Several factors may explain these findings: firstly, the elderly patients in the included papers showed higher incidence of hypertension, diabetes, coronary-artery and lung disease and ASA score III/IV, which define a higher frailty background. Moreover, older people tend to suffer more from weight loss with an increased vulnerability and a weakened functional reserve [4]. Secondly, several studies have stated that age and a higher ASA score are not strongly associated with the occurrence of DGE, POPF, post-operative bleeding and abdominal infection, and these last seem to be linked to technical issues [29,30]. Finally, functional impairment and comorbidities predispose elderly patients to a higher incidence of major non-surgical complications after a pancreaticoduodenectomy which leads to a longer hospitalization and a higher mortality rate [4,10,31]. 

Indeed, this study showed a significantly higher mortality rate in the MIS*e* group (4%, range: 1.6–7.4%) compared with the MIS*y* group (1%, range: 0–2.4%); the hospital stay was longer for older patients than for younger, at 17.5 days and 13.8 days, respectively, as well. No difference was outlined in the rate of readmission to hospital. However, this evidence in elderly patients is still discordant and debated. 

Elderly patients should not be excluded from surgical resection because of their advanced age. To partially overcome and mitigate morbidities related to pancreatic surgery, extensive efforts should be made for patient selection, preoperative frailty assessment and earlier postoperative rehabilitation. Nevertheless, patient prehabilitation becomes pivotal in improving the fitness levels of the elderly before surgery, and to increasing the number of patients able to receive adjuvant treatments within an appropriate time [2,32]. Moreover, the well-known advantages of MIS should be taken into account while evaluating quality rather than the quantity of life of patients with pancreatic head lesions [33]. The real added value of MIS in older patients is still uncertain. MIS is undoubtedly associated with faster recovery, less post-operative pain and immobility; however, the main factors in determining the speed of recovery are the healing of anastomosis and the resumption of gastrointestinal function [34].

As a minor endpoint of our meta-analysis, oncological safety was also evaluated: no differences in terms of mean number of harvested lymphnodes and in R0 resection rates were found. The mean number of harvested lymphnodes was 14 in both groups and the R0 rates were similar: 96% in MIS*e* and 98% in MIS*y*. We underline that there was an intrinsic selection bias in the baseline patients’ characteristics and in the indication for operative procedures in the included studies. However, our oncological findings should be taken into account because there were no differences in terms of tumor size, T-stage and distribution of both benign and malignant diseases between the two groups (Table 2). Moreover, about 80% of patients had pancreatic duct adenocarcinoma. Several authors have demonstrated the oncologic efficacy of MIS with a higher number of harvested lymphnodes and comparable R0 resection rates; nonetheless, without a clear advantage in overall survival, tumor biology drives patients’ prognoses [35,36].

To our knowledge this is the first meta-analysis that evaluates these comparisons. Despite the high quality of the included papers, there are several limitations concerning our meta-analysis. All the included studies were retrospective and involved some selection bias; however, we always applied a random-effect model to limit and mitigate the influence of variables’ heterogeneity among studies. Moreover, some continuous outcomes were reported in an unclear manner and, as a result, some of them were dismissed from the analysis: the missing data can be inferred from Table 1 and Table 2. Furthermore, a subgroup analysis between the laparoscopic and robotic approaches was not conducted due to the limited number of included studies. This could mitigate and blend some of the emerging advantages of robotic surgery over the laparoscopic approach. In addition, there is still not a clear and shared definition of elderly patients, and the cut-off point ranges from 65 to 75 years between the included studies; therefore, this could lead to inaccurate conclusions. Finally, uneven surgical procedures, the different surgical modalities and the different learning curves of different surgeons, as well as the regional differences (all papers except one came from China), may potentially contribute to the heterogeneity of the final results. However, for the first time, our systematic review summarizes most of the available evidence in comparing outcomes of MIPD in elderly and younger patients. 

## 5. Conclusions

MIPD seems to be relatively safe and feasible; however, it results in slightly higher mortality, lung complications and major morbidity rates in elderly patients, who potentially represent the group of patients that may benefit the most from reduced surgical and immunological stress. However, the real additional value of MIS in this frailty subset of patients needs to be further investigated. In particular, robotic surgery could play a central role in the reduction of surgical complications by overcoming the inherent well-known shortcomings of the laparoscopic approach. Therefore, pancreatic resection should not be denied to elderly patients. More efforts should be made to improve patient selection and pre-habilitation; clear guidelines and concerns about the quality of life should be developed and scrutinized in order to lower the risk of under-treatment in elderly patients. 

## Figures and Tables

**Table 1:** Summary of studies included in the meta-analysis.

n.	Author	Region	Year	Study Period	Study Design and Cut-Off Age	Sample Size		Age (Years)		Groups	MINORS (Quality)
						MIS*e*	MIS*y*	MIS*e*	MIS*y*		
1	Buchs [21]	USA	2010	2007–2010	OCS (P)—70	15	26	76.8	56.3	RPDe vs. RPDy	22
2	Liang [10]	China	2019	2015–2018	OCS (R)—70	27	55	74.0	59.0	LPDe vs. LPDy	22
3	Cai [22]	China	2020	2012–2019	OCS (R)—70	51	96	75.2	56.1	LPDe vs. LPDy	21
4	Hendi [23]	China	2020	2012–2017	OCS (P)—75	61	176	75.7	55.7	LPDe vs. LPDy	23
5	Ke [24]	China	2020	2015–2019	OCS (R)—65	75	225	>65	<65	LPDe vs. LPDy	22
6	Liu [4]	China	2020	2018–2019	OCS (R)—75	77	354	77.0	57.9	RPDe vs. RPDy	23
7	Tan [25]	China	2020	2015–2017	OCS (R)—70	56	84	75.2	60.7	LPDe vs. LPDy	23

OCS: observational clinical study; P: prospectively collected data; R: retrospectively collected data; e: elderly; y: young; MIS: minimally invasive surgery; LPD: laparoscopic pancreaticoduodenectomy; RPD: robotic pancreaticoduodenectomy.

**Table 2:** General and patient characteristics.

	MIS*e*	MIS*y*	Studies (n)
Total patients included	362	1016	1378 (7)
Age (years)	75.7	57.6	6
Male/Female (%)	214/148 (59.1)	590/426 (58.1)	7
BMI	23.0	23.6	7
Total Bilirubin (mmol/L)	134.0	146.7	2
CA 19.9 U/ml	187.5	133.6	2
Pre-op Biliary drainage (%)	16 (18.0)	42 (17.0)	2
ASA I/II (%)	213 (61.0)	865 (87.0)	6
ASA III/IV (%)	134 (39.0)	125 (13.0)	6
Overall Comorbidity (%)	112 (59.0)	206 (33.0)	3
Hypertension (%)	95 (42.0)	186 (24.0)	4
CAD (%)	28 (10.0)	56 (6.0)	4
Diabetes (%)	30 (18.0)	94 (16.0)	3
Neurological disease (%)	4 (4.0)	2 (1.0)	2
Lung comorbidity (%)	10 (6.0)	25 (4.0)	3
Benign disease (%)	56 (15.0)	225 (22.0)	7
Malignant disease (%)	306 (85.0)	791 (78.0)	7
Maximum tumor diameter (cm)	2.66	2.66	5
Whipple procedure (%)	308 (99.0)	908 (99.0)	6
Pylorus-preserving procedure (%)	3 (1.00)	12 (1.00)	6
TNM 1a (%)	7 (11.0)	9 (8.00)	2
TNM 1b (%)	33 (53.0)	61 (53.0)	2
TNM 2a (%)	10 (16.0)	23 (20.0)	2
TNM 2b (%)	8 (13.0)	19 (16.0)	2
TNM 3 (%)	4 (6.0)	4 (3.0)	2
TNM 4 (%)	0	0	2

Continuous variables are expressed as mean; MIS: minimally invasive surgery; e: elderly; y: young; BMI: body mass index, CA 19.9: carbohydrate antigen 19.9; ASA: American Society of Anesthesiologists; CAD: coronary artery disease; TNM: staging system; n: number.

**Table 3:** Technical and post-operative outcomes.

Surgical Outcome	Type of Surgery	Observations (n)	Mean or %	Studies Included (n)	*p*-Value
Operating time (min)	MIS*e*	362	354.06	7	0.07
	MIS*y*	1016	354.49		
Blood loss (mL)	MIS*e*	362	237.49	7	0.003
	MIS*y*	1016	191.27		
Intra-op Transfusion rate	MIS*e*	34/223	15.0%	4	0.04
	MIS*y*	51/689	7.0%		
Conversion to Open rate	MIS*e*	10/226	4.0%	5	0.86
	MIS*y*	24/615	4.0%		
Reoperation rate	MIS*e*	15/362	4.0%	7	0.75
	MIS*y*	40/1016	4.0%		
Peri-op Mortality rate	MIS*e*	13/362	4.0%	7	0.02
	MIS*y*	14/1016	1.0%		
Overall Complication rate	MIS*e*	96/204	47.0%	4	0.08
	MIS*y*	229/652	35.0%		
Clavien–Dindo I/II rate	MIS*e*	112/211	53.0%	4	0.86
	MIS*y*	238/589	40.0%		
Clavien–Dindo ≥ III rate	MIS*e*	38/211	18.0%	4	0.02
	MIS*y*	64/589	11.0%		
POPF grade > A rate	MIS*e*	49/362	14.0%	7	0.24
	MIS*y*	110/1016	11.0%		
Abdominal Collection rate	MIS*e*	21/155	14.0%	3	0.26
	MIS*y*	50/505	10.0%		
Biliary Leakage rate	MIS*e*	13/279	5.0%	5	0.22
	MIS*y*	30/877	3.0%		
Post-op Bleeding rate	MIS*e*	24/287	8.0%	6	0.22
	MIS*y*	53/791	7.0%		
DGE rate	MIS*e*	33/301	11.0%	6	0.20
	MIS*y*	76/840	9.0%		
Lung Morbidity rate	MIS*e*	20/167	12.0%	3	0.02
	MIS*y*	42/605	7.0%		
R0-margin rate	MIS*e*	187/195	96.0%	4	0.34
	MIS*y*	401/411	98.0%		
N. harvested lymphnodes	MIS*e*	270	14.4	5	0.98
	MIS*y*	636	14.3		
Readmission rate	MIS*e*	12/235	5.0%	4	0.67
	MIS*y*	44/718	6.0%		
Hospital stay (days)	MIS*e*	362	17.5	7	0.003
	MIS*y*	1016	13.8		

Continuous variables are expressed as mean; MIS: minimally invasive surgery; e: elderly; y: young; POPF: post-operative pancreatic fistula; DGE: delayed gastric empty; n: number; op: operative.

## Data Availability

Not applicable.

## Supplementary Materials

The following are available online at https://www.mdpi.com/article/10.3390/cancers16020323/s1, Supplementary File S1: PRISMA checklist [37], Supplementary File S2: MOOSE checklist, Supplementary File S3: Search strategy, Supplementary File S4: Publication bias and Supplementary File S5: Individual studies’ characteristics.
