# Supplementary material for: Minimally Invasive Pancreaticoduodenectomy in Elderly versus Younger Patients: A Meta-Analysis
Source: Cancers (Basel). 2024 Jan 11;16(2):323. doi: 10.3390/cancers16020323 (PMC10813942; doi:10.3390/cancers16020323)
Supplement: Supplementary file 1 [file cancers-16-00323-s001.zip › Supplementary file S3 - Search History.pdf]

Search number,Query,Sort By,Filters,Search Details,Results,Time
13,((pancreaticoduodenectomy) AND (laparoscopic OR robotic OR minimally invasive)) AND (elderly OR older),,,"(""pancreaticoduodenectomy""[MeSH Terms] OR ""pancreaticoduodenectomy""[All Fields] OR ""pancreaticoduodenectomies""[All Fields]) AND (""laparoscopes""[MeSH Terms] OR ""laparoscopes""[All Fields] OR ""laparoscope""[All Fields] OR ""laparoscopical""[All Fields] OR ""laparoscopically""[All Fields] OR ""laparoscopics""[All Fields] OR ""laparoscopy""[MeSH Terms] OR ""laparoscopy""[All Fields] OR ""laparoscopic""[All Fields] OR (""robot""[All Fields] OR ""robot s""[All Fields] OR ""robotically""[All Fields] OR ""robotics""[MeSH Terms] OR ""robotics""[All Fields] OR ""robotic""[All Fields] OR ""robotization""[All Fields] OR ""robotized""[All Fields] OR ""robots""[All Fields]) OR (""minimally""[All Fields] AND (""invasibility""[All Fields] OR ""invasible""[All Fields] OR ""invasion""[All Fields] OR ""invasions""[All Fields] OR ""invasive""[All Fields] OR ""invasively""[All Fields] OR ""invasiveness""[All Fields] OR ""invasives""[All Fields] OR ""invasivity""[All Fields]))) AND (""aged""[MeSH Terms] OR ""aged""[All Fields] OR ""elderly""[All Fields] OR ""elderlies""[All Fields] OR ""elderly s""[All Fields] OR ""elderlys""[All Fields] OR (""older""[All Fields] OR ""olders""[All Fields]))",612,09:49:18
12,((pancreaticoduodenectomy) AND (laparoscopic OR robotic)) AND (elderly OR older),,,"(""pancreaticoduodenectomy""[MeSH Terms] OR ""pancreaticoduodenectomy""[All Fields] OR ""pancreaticoduodenectomies""[All Fields]) AND (""laparoscopes""[MeSH Terms] OR ""laparoscopes""[All Fields] OR ""laparoscope""[All Fields] OR ""laparoscopical""[All Fields] OR ""laparoscopically""[All Fields] OR ""laparoscopics""[All Fields] OR ""laparoscopy""[MeSH Terms] OR ""laparoscopy""[All Fields] OR ""laparoscopic""[All Fields] OR (""robot""[All Fields] OR ""robot s""[All Fields] OR ""robotically""[All Fields] OR ""robotics""[MeSH Terms] OR ""robotics""[All Fields] OR ""robotic""[All Fields] OR ""robotization""[All Fields] OR ""robotized""[All Fields] OR ""robots""[All Fields])) AND (""aged""[MeSH Terms] OR ""aged""[All Fields] OR ""elderly""[All Fields] OR ""elderlies""[All Fields] OR ""elderly s""[All Fields] OR ""elderlys""[All Fields] OR (""older""[All Fields] OR ""olders""[All Fields]))",553,09:39:03
9,((pancreaticoduodenectomy) AND (minimally invasive)) AND (elderly),,,"(""pancreaticoduodenectomy""[MeSH Terms] OR ""pancreaticoduodenectomy""[All Fields] OR ""pancreaticoduodenectomies""[All Fields]) AND (""minimally""[All Fields] AND (""invasibility""[All Fields] OR ""invasible""[All Fields] OR ""invasion""[All Fields] OR ""invasions""[All Fields] OR ""invasive""[All Fields] OR ""invasively""[All Fields] OR ""invasiveness""[All Fields] OR ""invasives""[All Fields] OR ""invasivity""[All Fields])) AND (""aged""[MeSH Terms] OR ""aged""[All Fields] OR ""elderly""[All Fields] OR ""elderlies""[All Fields] OR ""elderly s""[All Fields] OR ""elderlys""[All Fields])",232,09:33:26
8,((pancreaticoduodenectomy) AND (elderly)) AND (laparoscopic),,,"(""pancreaticoduodenectomy""[MeSH Terms] OR ""pancreaticoduodenectomy""[All Fields] OR ""pancreaticoduodenectomies""[All Fields]) AND (""aged""[MeSH Terms] OR ""aged""[All Fields] OR ""elderly""[All Fields] OR ""elderlies""[All Fields] OR ""elderly s""[All Fields] OR ""elderlys""[All Fields]) AND (""laparoscopes""[MeSH Terms] OR ""laparoscopes""[All Fields] OR ""laparoscope""[All Fields] OR ""laparoscopical""[All Fields] OR ""laparoscopically""[All Fields] OR ""laparoscopics""[All Fields] OR ""laparoscopy""[MeSH Terms] OR ""laparoscopy""[All Fields] OR ""laparoscopic""[All Fields])",463,09:32:50
7,((pancreaticoduodenectomy) AND (elderly)) AND (robot),,,"(""pancreaticoduodenectomy""[MeSH Terms] OR ""pancreaticoduodenectomy""[All Fields] OR ""pancreaticoduodenectomies""[All Fields]) AND (""aged""[MeSH Terms] OR ""aged""[All Fields] OR ""elderly""[All Fields] OR ""elderlies""[All Fields] OR ""elderly s""[All Fields] OR ""elderlys""[All Fields]) AND (""robot""[All Fields] OR ""robot s""[All Fields] OR ""robotically""[All Fields] OR ""robotics""[MeSH Terms] OR ""robotics""[All Fields] OR ""robotic""[All Fields] OR ""robotization""[All Fields] OR ""robotized""[All Fields] OR ""robots""[All Fields])",171,09:32:29
6,((pancreaticoduodenectomy) AND (elderly)) AND (young),,,"(""pancreaticoduodenectomy""[MeSH Terms] OR ""pancreaticoduodenectomy""[All Fields] OR ""pancreaticoduodenectomies""[All Fields]) AND (""aged""[MeSH Terms] OR ""aged""[All Fields] OR ""elderly""[All Fields] OR ""elderlies""[All Fields] OR ""elderly s""[All Fields] OR ""elderlys""[All Fields]) AND (""young""[All Fields] OR ""youngs""[All

Fields])",560,09:31:40
5,(pancreaticoduodenectomy) AND (elderly),,,"(""pancreaticoduodenectomy""[MeSH Terms] OR ""pancreaticoduodenectomy""[All Fields] OR ""pancreaticoduodenectomies""[All Fields]) AND (""aged""[MeSH Terms] OR ""aged""[All Fields] OR ""elderly""[All Fields] OR ""elderlies""[All Fields] OR ""elderly s""[All Fields] OR ""elderlys""[All Fields])","7,602",09:31:21
4,minimally invasive pancreaticoduodenectomy,,,"""minimally""[All Fields] AND (""invasibility""[All Fields] OR ""invasible""[All Fields] OR ""invasion""[All Fields] OR ""invasions""[All Fields] OR ""invasive""[All Fields] OR ""invasively""[All Fields] OR ""invasiveness""[All Fields] OR ""invasives""[All Fields] OR ""invasivity""[All Fields]) AND (""pancreaticoduodenectomy""[MeSH Terms] OR ""pancreaticoduodenectomy""[All Fields] OR ""pancreaticoduodenectomies""[All Fields])",608,09:30:23
3,robotic pancreaticoduodenectomy,,,"(""robot""[All Fields] OR ""robot s""[All Fields] OR ""robotically""[All Fields] OR ""robotics""[MeSH Terms] OR ""robotics""[All Fields] OR ""robotic""[All Fields] OR ""robotization""[All Fields] OR ""robotized""[All Fields] OR ""robots""[All Fields]) AND (""pancreaticoduodenectomy""[MeSH Terms] OR ""pancreaticoduodenectomy""[All Fields] OR ""pancreaticoduodenectomies""[All Fields])",483,09:30:03
2,laparoscopic pancreaticoduodenectomy,,,"(""laparoscopes""[MeSH Terms] OR ""laparoscopes""[All Fields] OR ""laparoscope""[All Fields] OR ""laparoscopical""[All Fields] OR ""laparoscopically""[All Fields] OR ""laparoscopics""[All Fields] OR ""laparoscopy""[MeSH Terms] OR ""laparoscopy""[All Fields] OR ""laparoscopic""[All Fields]) AND (""pancreaticoduodenectomy""[MeSH Terms] OR ""pancreaticoduodenectomy""[All Fields] OR ""pancreaticoduodenectomies""[All Fields])","1,097",09:29:49
1,pancreaticoduodenectomy,,,"""pancreaticoduodenectomy""[MeSH Terms] OR ""pancreaticoduodenectomy""[All Fields] OR ""pancreaticoduodenectomies""[All Fields]","13,213",09:29:24
