# Supplementary material for: Minimally Invasive Pancreaticoduodenectomy in Elderly versus Younger Patients: A Meta-Analysis
Source: Cancers (Basel). 2024 Jan 11;16(2):323. doi: 10.3390/cancers16020323 (PMC10813942; doi:10.3390/cancers16020323)

Funnel Plot

Figure S1 - Operating time (min)

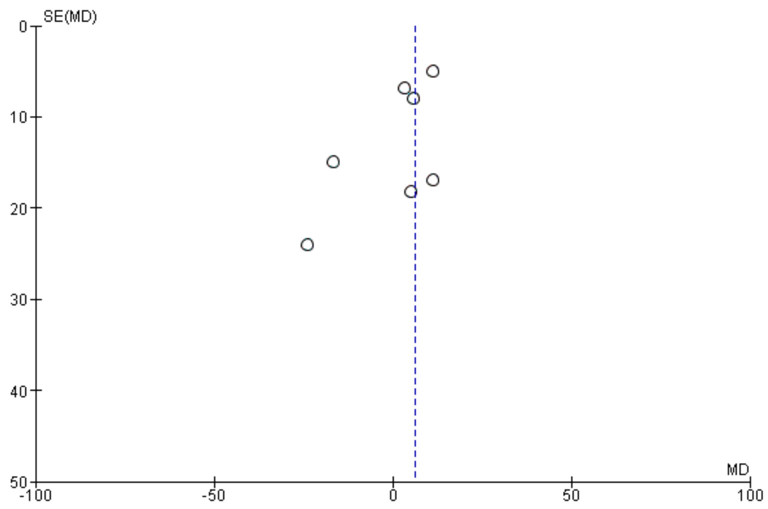

Figure S2 - Intraoperative blood loss (ml)

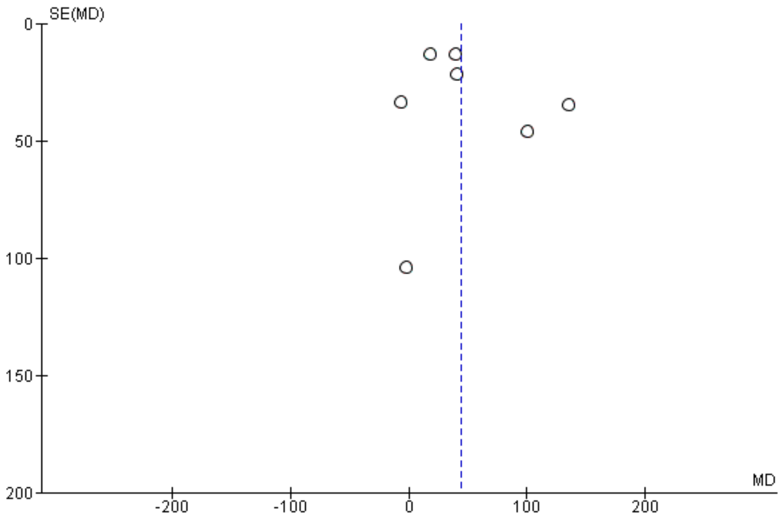

Figure S3 - Intraoperative Transfusion rate

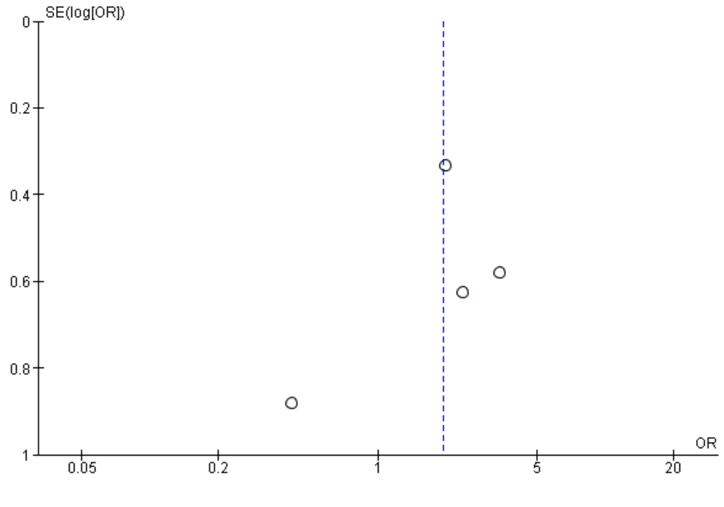

Figure S4 - Conversion to open rate

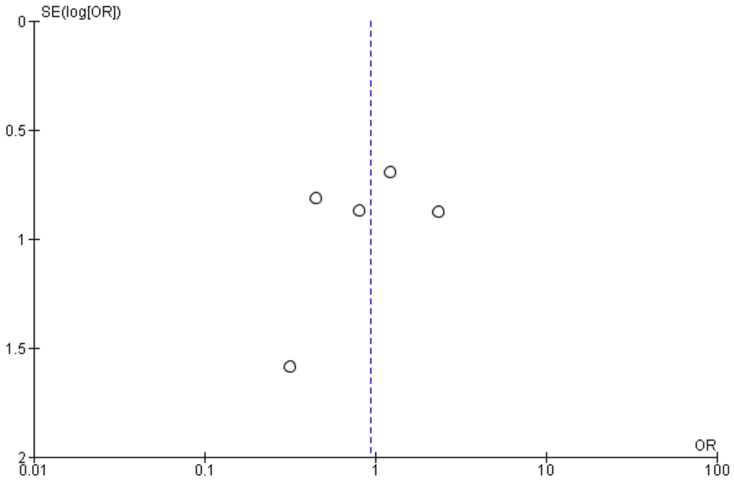

Figure S5 - Reoperation rate

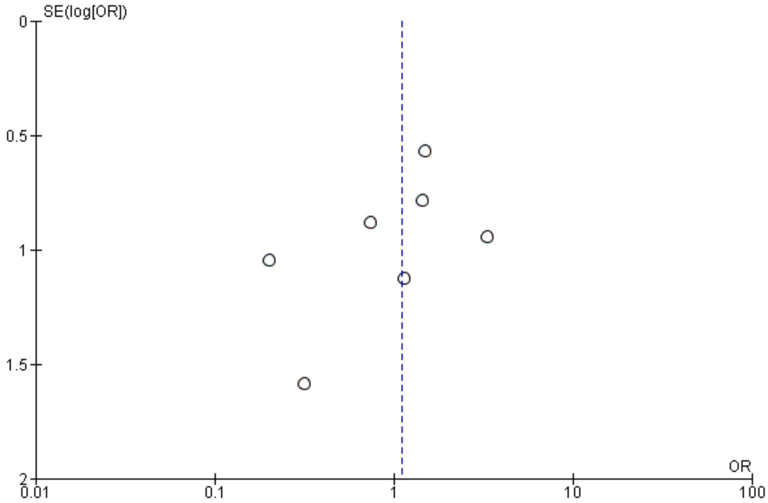

Figure S6 - Perioperative mortality rate

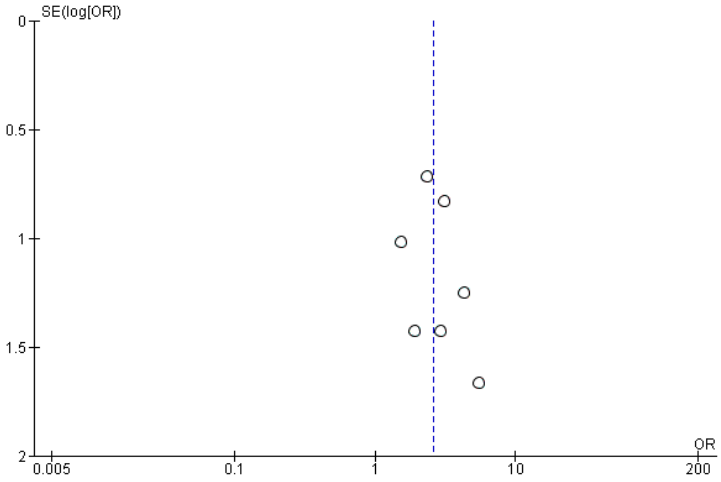

Figure S7 - Overall Complication rate

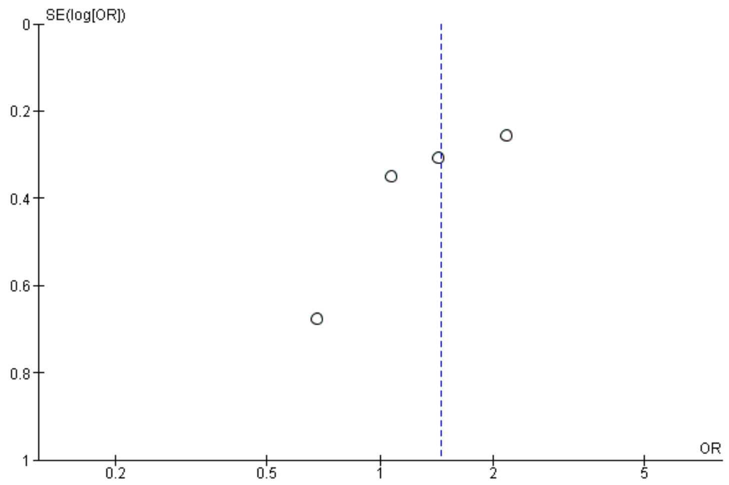

Figure S8 - Clavien-Dindo I/II rate

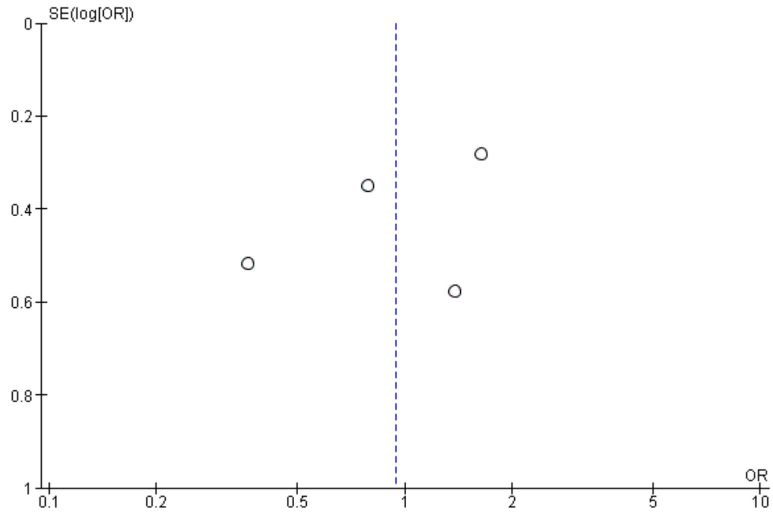

Figure S9 - Clavien-Dindo ≥ III rate

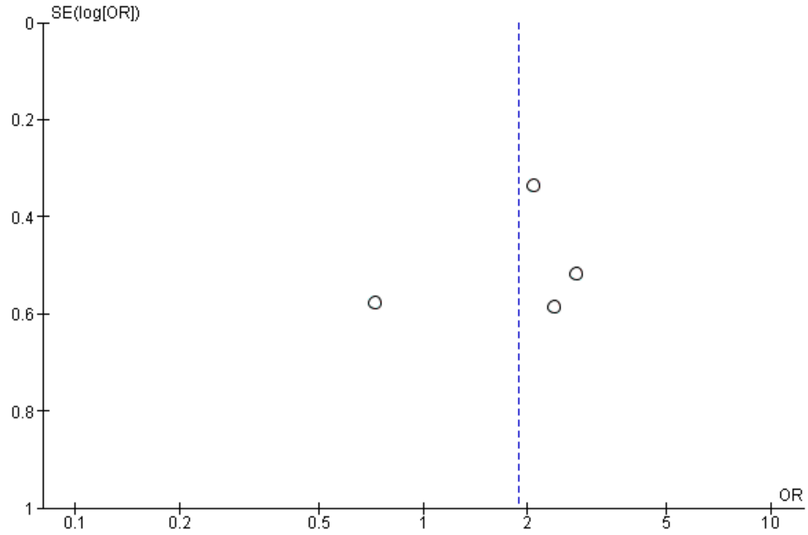

Figure S10 - POPF grade > A rate

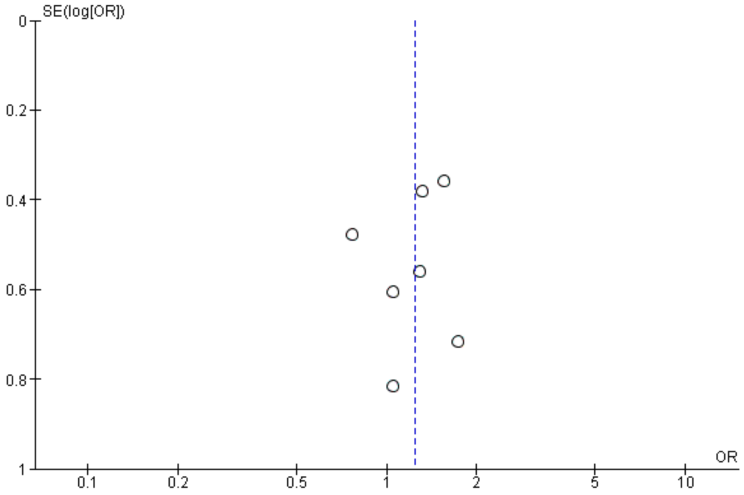

Figure S11 - Abdominal Collection rate

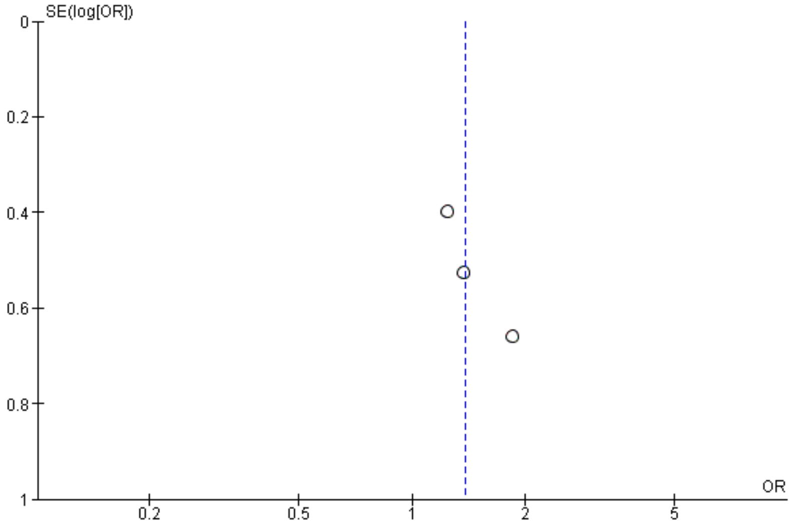

Figure S12 - Biliary Leakage rate

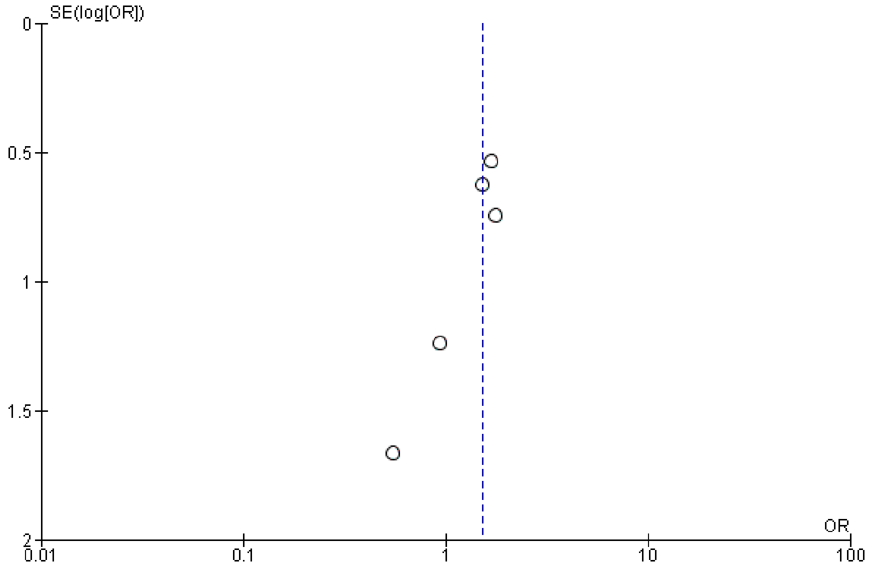

Figure S13 - Postoperative Bleeding rate

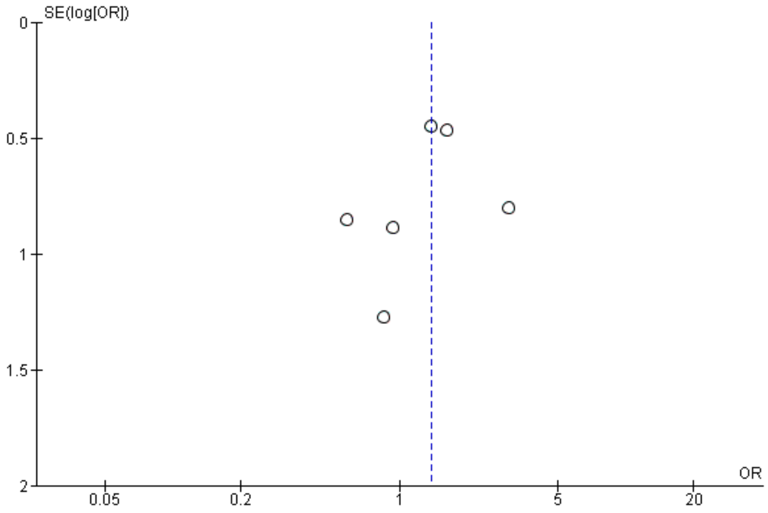

Figure S14 - Delayed Gastric Empty rate

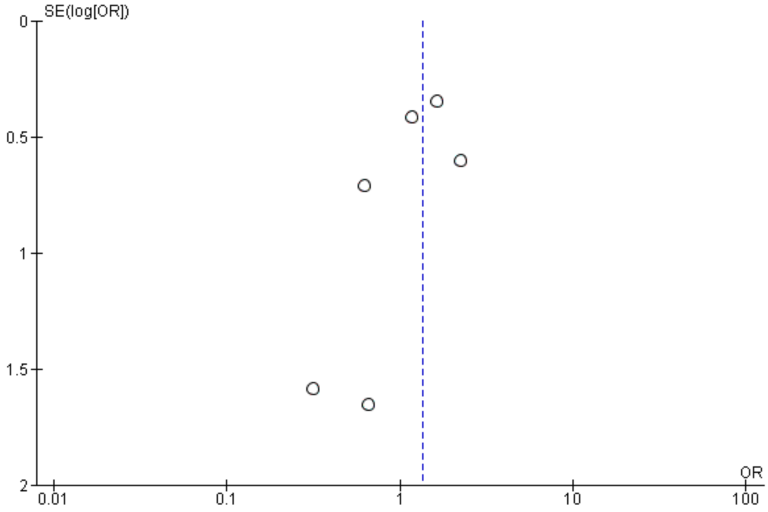

Figure S15 - Lung Morbidity rate

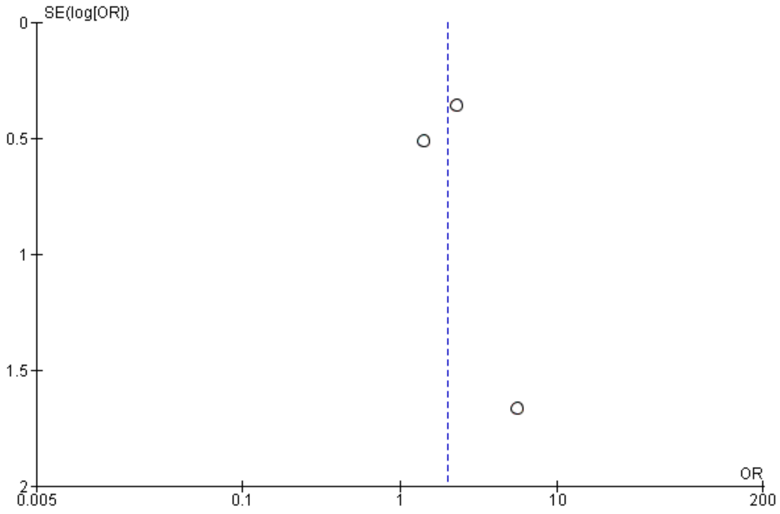

Figure S16 - R0-margin rate

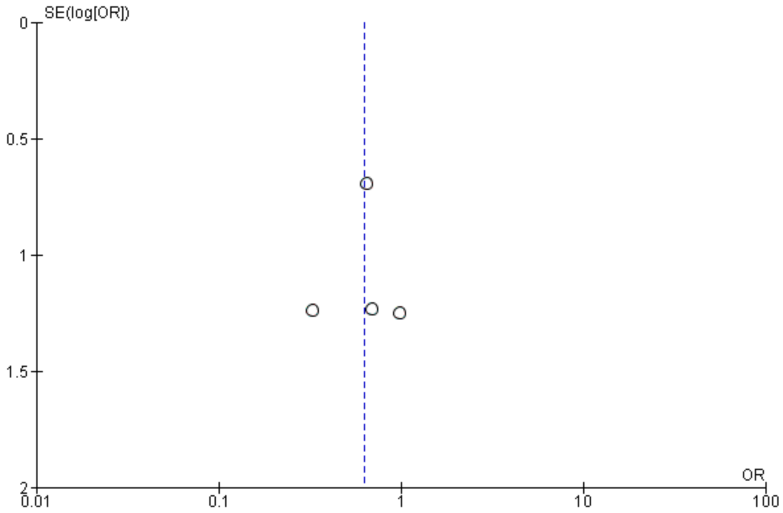

Figure S17 - Number of Harvested Lymphnodes

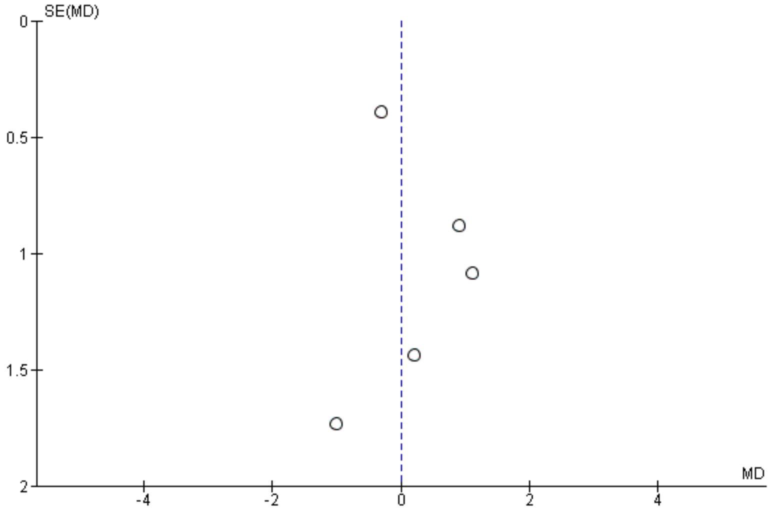

Figure S18 - Readmission rate

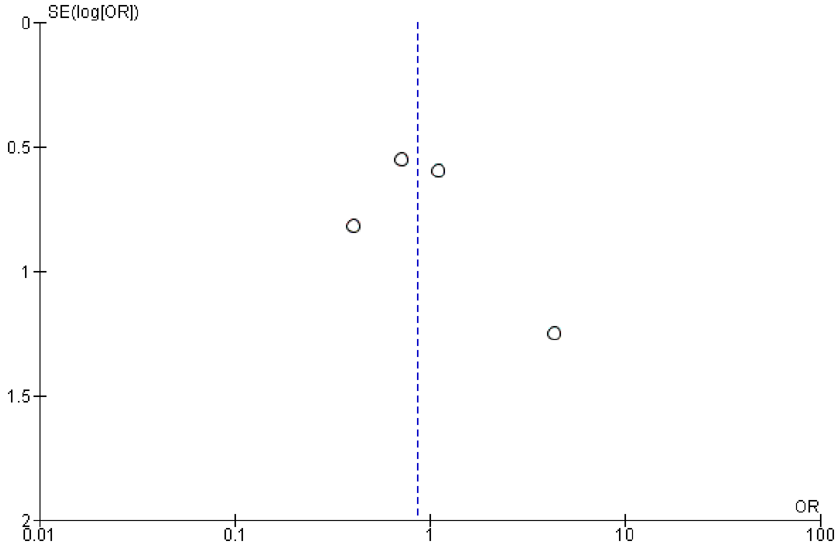

Figure S19 - Lenght of Hospital Stay (days)

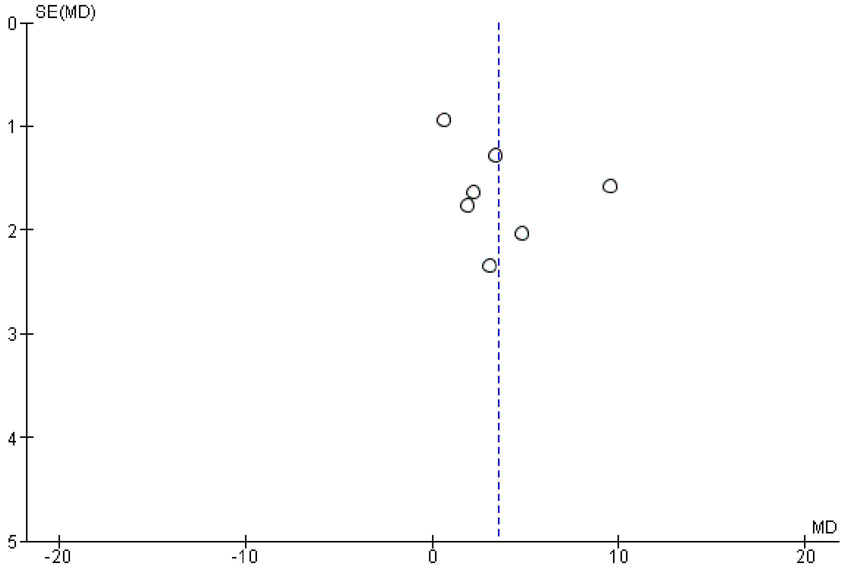

Supplement: Supplementary file 1 [file cancers-16-00323-s001.zip › Supplementary file S4 - Publication Bias Funnel Plot.pdf]
