# Supplementary material for: Minimally Invasive Pancreaticoduodenectomy in Elderly versus Younger Patients: A Meta-Analysis
Source: Cancers (Basel). 2024 Jan 11;16(2):323. doi: 10.3390/cancers16020323 (PMC10813942; doi:10.3390/cancers16020323)
Supplement: Supplementary file 1 [file cancers-16-00323-s001.zip › Supplementary file S5 - Individual studies' characteristics.pdf]

n.	Author	Age (range or SD)	*p-value*	Gender (M/F)	*p-value*	BMI	*p-value*	Total Bilirubin (mmol/L)	*p-value*	CA 19.9 U/ml	*p-value*	Pre-op Biliary drainage(%)	*p-value*
1	Buchs [21]	76.8 (70-82) vs 56.3 (24-69)	**<0.0001**	8/7 vs 12/14	0.75	27.5 vs 28.2	0.69	NA	-	NA	-	20 vs 34.6	0.48
2	Liang [10]	74.0 (4.0) vs 59.0 (9.0)	**<0.0001**	16/11 vs 30/25	0.81	22.0 vs 22.0	0.41	NA	-	NA	-	NA	-
3	Cai [22]	75.2 (3.9) vs 56.1 (9.4)	**<0.0001**	30/21 vs 61/35	0.57	21.3 vs 21.4	0.81	134 vs 146.7	0.09	187.5 vs 133.6	0.46	NA	-
4	Hendi [23]	75.7 (4.9) vs 55.7 (10.8)	**<0.0001**	37/24 vs 107/69	0.89	22.2 vs 23.1	0.78	NA	-	NA		NA	-
5	Ke [24]	>65 (NA) vs <65 (NA)	NA	45/30 vs 120/105	0.32	22.8 vs 23.2	0.34	110.9 vs 105.3	0.49	92.1 vs 86.3	0.26	26.7 vs 28.4	0.77
6	Liu [4]	77.0 (3) vs 57.9 (12.4)	**<0.0001**	47/30 vs 209/145	0.75	22.8 vs 23.8	**0.01**	NA	-	NA	-	NA	-
7	Tan [25]	75.2 (4.4) vs 60.7 (7.5)	**<0.0001**	33/23 vs 51/33	0.83	22.2 vs 23.3	**0.02**	NA	-	NA	-	NA	-

n.	Author	*ASA I/II (%)*	*p-value*	ASA III/IV (%)	*p-value*	Overall Comorbidity (%)	*p-value*	Hypertension (%)	*p-value*	CAD (%)	*p-value*	Diabetes	*p-value*
1	Buchs [21]	NA	-	NA	-	NA	-	53.3 vs 53.8	1.00	20 vs 11.5	0.65	20 vs 15.4	0.69
2	Liang [10]	70.4 vs 98.2	**<0.0001**	29.6 vs 1.8	**<0.0001**	NA	-	NA	-	NA	-	NA	-
3	Cai [22]	70.6 vs 75	0.57	29.4 vs 25	0.57	62.7 vs 36.5	**0.002**	NA	-	NA	-	NA	-
4	Hendi [23]	82 vs 96.6	**0.001**	18 vs 3.4	**0.001**	68.9 vs 34.6	**0.0001**	41 vs 22.2	**0.007**	6.6 vs 1.1	**0.04**	NA	-
5	Ke [24]	76 vs 96	**<0.0001**	24 vs 4	**<0.0001**	NA	-	17.3 vs 10.7	0.13	12 vs 2.2	0.33	12 vs 9.8	0.58
6	Liu [4]	66.2 vs 99.7	**<0.0001**	33.8 vs 0.3	**<0.0001**	49.3 vs 28.2	NA	63.6 vs 30.8	**<0.0001**	22.1 vs 13	0.16	23.3 vs 19.2	0.41
7	Tan [25]	66.1 vs 78.6	0.10	33.9 vs 21.4	0.10	NA	-	NA	-	NA	-	NA	-

n.	Author	*Lung disease (%)*	*p-value*	*Benign disease (%)*	*p-value*	*Malignant disease (%)*	*p-value*	*Max tumor diameter (%)*	*p-value*	*Whipple procedure (%)*	*p-value*	*Pylorus preserving (%)*	*p-value*
1	Buchs [21]	0 vs 11.5	0.28	26.7 vs 23.1	1.00	73.3 vs 76.9	1.00	NA	-	80 vs 53.8	0.18	20 vs 46.2	0.18
2	Liang [10]	NA	-	11.1 vs 34.5	**0.03**	88.9 vs 65.5	**0.03**	2.6 vs 2.8	0.55	100 vs 100	1.00	0 vs 0	1.00
3	Cai [22]	NA	-	0 vs0	1.00	100 vs 100	1.00	3.1 vs 3.3	0.30	NA	-	NA	-
4	Hendi [23]	NA	-	64 vs 20	0.76	36 vs 80	0.76	2.4 vs 2.3	0.86	100 vs 100	1.00	0 vs 0	1.00
5	Ke [24]	2.7 vs 0.44	0.16	5.3 vs 25.3	**0.001**	94.7 vs 74.7	**0.001**	2.3 vs 2.6	0.42	100 vs 100	1.00	0 vs 0	1.00
6	Liu [4]	10.4 vs 5.9	0.16	7.2 vs 19.2	0.11	92.2 vs 80.8	0.11	NA	-	100 vs 100	1.00	0 vs 0	1.00
7	Tan [25]	NA	-	33.3 vs 21.4	1.00	66.7 vs 78.6	1.00	2.9 vs 2.3		100 vs 100	1.00	0 vs 0	1.00

n.	Author	*TMN 1a (%)*	*p-value*	*TMN 1b (%)*	*p-value*	*TMN 2a (%)*	*p-value*	*TMN 2b (%)*	*p-value*	*TMN 3 (%)*	*p-value*	*TMN 4 (%)*	*p-value*
1	Buchs [21]	9.1 vs 5	1.00	18.2 vs 20	1.00	27.3 vs 35	1.00	27.3 vs 35	1.00	18.2 vs 5	0.25	0 vs 0	-
2	Liang [10]	NA	-	NA	-	NA	-	NA	-	NA	-	NA	-
3	Cai [22]	11.8 vs 8.3	0.59	60.8 vs 59.3	0.59	13.7 vs 16.7	0.59	9.8 vs 12.5	0.59	3.9 vs 3.2	0.59	0 vs 0	-
4	Hendi [23]	NA	-	NA	-	NA	-	NA	-	NA	-	NA	-
5	Ke [24]	NA	-	NA	-	NA	-	NA	-	NA	-	NA	-
6	Liu [4]	NA	-	NA	-	NA	-	NA	-	NA	-	NA	-
7	Tan [25]	NA	-	NA	-	NA	-	NA	-	NA	-	NA	-
